# Tumor Microenvironment Dynamics of Triple-Negative Breast Cancer Under Radiation Therapy

**DOI:** 10.3390/ijms26062795

**Published:** 2025-03-20

**Authors:** Suryakant Niture, Subhajit Ghosh, Jerry Jaboin, Danushka Seneviratne

**Affiliations:** Department of Radiation Oncology, Stephenson Cancer Center, Oklahoma University, Oklahoma City, OK 73104, USA

**Keywords:** triple-negative breast cancer, tumor microenvironment, radiation therapy, radioresistance, immune response

## Abstract

Triple-negative breast cancer (TNBC) is an aggressive subtype of breast cancer characterized by the absence of estrogen receptors (ER), progesterone receptors (PR), and HER2 expression. While TNBC is relatively less common, accounting for only 10–15% of initial breast cancer diagnosis, due to its aggressive nature, it carries a worse prognosis in comparison to its hormone receptor-positive counterparts. Despite significant advancements in the screening, diagnosis, and treatment of breast cancer, TNBC remains an important public health burden. Following treatment with chemotherapy, surgery, and radiation, over 40% of TNBC patients experience relapse within 3 years and achieve the least benefit from post-mastectomy radiation. The tumor microenvironment environment (TME) is pivotal in TNBC initiation, progression, immune evasion, treatment resistance, and tumor prognosis. TME is a complex network that consists of immune cells, non-immune cells, and soluble factors located in the region adjacent to the tumor that modulates the therapeutic response differentially between hormone receptor-positive breast cancer and TNBC. While the mechanisms underlying the radiation resistance of TNBC remain unclear, the immunosuppressive TME of TNBC has been implicated in chemotherapeutic resistance. Radiation therapy (RT) is known to alter the TME; however, immune changes elicited by radiation are poorly characterized to date, and whether these immune changes contribute to radiation resistance remains unknown. This review delves into the distinct characteristics of the TNBC TME, explores how RT influences TME dynamics, and examines mechanisms underlying tumor radiosensitization, radioresistance, and immune responses.

## 1. Introduction

### 1.1. Breast Cancer Biological and Molecular Subtypes

Approximately one in eight women are diagnosed with breast cancer (BC) in the United States and BC remains a leading cause of cancer-related mortality worldwide [1,2]. There is considerable biological heterogeneity among different breast cancer subtypes, resulting in markedly varying clinical outcomes. The biological characterization of BC is based primarily on the expression of estrogen receptor (ER), progesterone receptor (PR), and human epidermal growth factor (HER2). The hormone receptor-positive (HR+) subtype expresses > 1% ER and/or PR, while the HER2-positive (HER2+) subtype demonstrates the abnormal amplification of the *ERBB2* gene and subsequent overexpression of the HER2 protein, whereas the triple-negative breast cancer (TNBC) subtype lacks the expression of all three markers [3,4]. Among the HER2-positive subtype, approximately 50% are classified as HR+, granting those distinct biological characteristics and clinical outcomes compared to the HR-negative HER2+ patients [5]. In addition to the hormone receptor-based biological classification system noted above, BC can be characterized into molecular subtypes based on gene expression patterns: Luminal A and B, HER2-enriched, and Basal-like (BL) breast cancer. Luminal A and B express ER-related genes corresponding to HR+/HER2 negative and HR+/HER2+ biological subtypes, respectively. The HER2-enriched subgroup lacks ER/PR expresses *ERBB2*-related genes and is associated with the HER2-positive subtype. The basal-like group primarily expresses epithelial-grown factor receptors (EGFR)-related genes and is associated with the TNBC subtype [6]. While most TNBCs coincide with the basal-like subtype, they are not necessarily synonymous and demonstrate up to 30% differences in gene expression patterns, Nevertheless, basal-like subtype and TNBC tend to demonstrate low *ER*/*PR*/*HER2* expression, and high expression *CK5*, *CK14*, *CAIX*, *CAVI*, *TP63*, *EGFR*, and *HER1* [4]. Regarding clinical outcomes, HR+/HER2 negative (−) subtype remains the best performer, with a 4-year survival rate of 92.5%, followed by HR+/HER+ subtype with a survival of 90%. HR negative (−)/HER2+ patients demonstrate a 4-year survival rate of 82%, while the worst outcomes are observed in the TNBC group with a survival rate of 77% [7].

### 1.2. Biological Heterogeneity of TNBC and Its Impact on Clinical Outcomes

Generally, before the advent of immunotherapy, TNBC patients were primarily treated with nonspecific neoadjuvant chemotherapy with taxanes or anthracyclines, followed by surgical resection, and often, radiation therapy. Only about 30% of TNBC patients demonstrate a pathologic complete response (PCR) to this chemotherapy regimen [8]. However, the recent addition of immunotherapy to neoadjuvant chemotherapy has increased PCR rates up to 60–65% [9]. Despite these therapeutic advances, TNBC remains difficult to control, demonstrating early disease recurrence, metastatic spread, and higher mortality rates compared to other BC sub-types [10]. TNBC cells exhibit a high mitotic rate, large nuclei with prominent nucleoli, and poorly defined cell borders reflecting an aggressive nature [11,12,13]. In addition, differences in the surrounding TME between the biological subtypes can also impact clinical outcomes, with recent findings identifying the unique TME of TNBC as a strong contributor to disease progression and invasion since TME consists of a variety of cells that include tumor-related immune cells, fibroblasts, and adipocytes [14]. The most famed of these genomic groups are the Lehmann’s clusters that were initially identified in 2011, which include immunomodulatory (IM), luminal androgen receptor (LAR), mesenchymal (M), two basal-like (BL1 and Bl2), and mesenchymal stem-like groups (MSLCs) [15]. This genomic classification of TNBC has demonstrated that the mutational landscape and TME can differ significantly among different TNBC patients and have distinct therapeutic implications [16,17]. Depending on the TNBC genomic division, potential therapeutic targets include DNA repair pathways, androgen receptor signaling (ARS), various kinase pathways, and immune-related targets [17]. For example, in the LAR group, mutations of AKT1 and BRCA1 contribute to PI3K pathway inhibitor sensitization and serve as an independent clinical prognostic factor. The basal-like immune-suppressed (BLIS) group is characterized by high genomic instability and the 3q 19-gene signature. The IM subtype demonstrates a higher expression of PD-L1 (≥1%) and the upregulation of Notch signaling, suggesting that immunotherapy and Notch inhibitors may benefit. Mesenchymal TNBC tumors demonstrate receptor tyrosine kinase (RTK)-RAS pathway enrichment, suggesting a benefit for treating with select kinase inhibitors [16]. Among the various biological BC subtypes, TNBC patients received the least benefit from post-mastectomy radiation suggesting that TNBC patients are more radiation-resistant [18,19]. Mechanisms underlying the radiation resistance of TNBC remain unclear, and the immunosuppressive TME within TNBC may contribute to radiation, and RT itself can dramatically alter the TME [20].

In this review, we discuss components of the tumor microenvironment environment (TME), the nature of TNBC-specific TME, the impact of radiation on the TNBC TME, and the role of RT in the modulation of TNBC immune response and potential mechanisms of radio-sensitization.

Methodology: Since TNBC accounts for only 13.6% of total BC cases, the molecular mechanisms related to the aggressive nature of TNBC and immune invasion in TME are poorly characterized. In this comprehensive review, we evaluated the updated literature on TNBC and addressed the nature of TME in TNBC and the mechanisms of immune invasion following radiation therapy. Our main focus in this review is how radiation therapy modulates the dynamics of immune invasion in TME and contributes to radiosensitization and radioresistance. Using up-to-date literature, we thoroughly reviewed the nature of TNBC subtypes, immune infiltration in TME, and the impact of radiation on the immune response within TNBC TME. Finally, we provided different scenarios of how radiation therapy contributes to TNBC treatment in the clinical setting.

## 2. TNBC TME

### 2.1. Nature of TME

**Figure 1:**
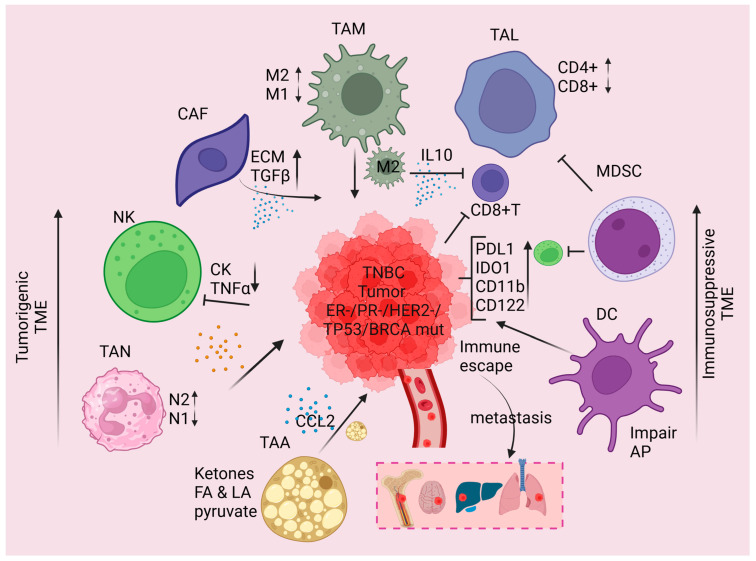
The schematic model represents the nature of TME of TNBC. Highly aggressive and metastatic TNBC tumors (ER-, PR-, HER2- and BRCA1/2 and TP53 mutants) received several pro-inflammatory prognosis factors from TAN-N1, TAM-M1, and chemokines and metabolic factors (ketones, fatty acid, and pyruvate) from TAA in TME. These unique conditions may favor the overexpression of PD-L1 and indoleamine 2, 3-dioxygenase 1 (IDO1) on the tumor surface modulate immune escapes from CD8+ T and DC cells. Upregulation of anti-inflammatory signaling inhibits CD8+ T cells, NK cell activity and creates immunosuppressive TME, leading to TNBC cell metastasis in the brain, lung, and liver. TAM—Tumor-associated macrophage; TAL—Tumor-associated lymphocyte; MDSCs—myeloid-derived suppressor cells; DC—dendritic cell; CAF—cancer-associated fibroblast; NK—Natural killer cell; TANs—Tumor-associated neutrophils; TAAs—tumor-associated adipocytes. The schematic model was generated using BioRender.

During cancer cell growth and tumor formation, the malignant tumor cells interact with the extracellular interstitium, creating a unique environment called TME. TME accounts for 10–20% of cancer cells and ~80% of other cells including immune cells, non-immune cells, and signaling factors. It is a complex network consisting of various cell signaling molecules (cytokines and chemokines), and the extracellular matrix (ECM), cancer-associated fibroblasts (CAFs), cancer-associated adipocytes (CAAs), tumor-infiltrating lymphocytes (TILs), tumor-associated macrophages (TAMs), tumor-associated neutrophils (TANs), natural killer (NK) cells, etc. [21,22] (Figure 1). Notably, neutrophils, myeloid-derived suppressor cells (MDSCs), TILs, and TAMs play dynamic roles in TME, and by infiltration and secretion of signaling factors indirectly modulated tumor growth, tumor metastasis, invasion, and treatment resistance [23]. The nature of TME can also be impacted by the patient’s health status, the location of the tumor, the stage, and the intrinsic features [24]. The extracellular pH of the TME is lower than normal tissue, and the abnormal vasculature of the TME can lead to limited blood flow to the tumor, leading to hypoxic conditions within certain parts of the tumor [25], and resultant metabolic heterogeneity [26]. Cancer cells can utilize soluble signaling factors and extracellular vesicles (which carry genetic material like miRNAs and ncRNAs) within the TME to transfer genetic information between the cancer cells to promote tumor growth, invasion, and metastasis [24,27,28,29]. Tumor cells can modulate the tumor immune microenvironment for their benefit. For instance, tumor cells can “steal” mitochondria from the surrounding immune cells to receive a metabolic boost, release lactate that can re-program the dendritic cells within the TME [30,31], and lead to the constitutive activation of the STING pathway to desensitize the immune system. Tumor cells promote immunosuppression within the TME via recruitment of MDSCs and immunosuppressive macrophages through the overexpression of cytidine deaminase and the overproduction of uridine diphosphate [32,33]. The number and the presence or absence of subsets of immune cells, immune memory, exhaustion state of leukocytes, and their functionality play dynamic roles in TME [14]. In an immunosuppressive TME, tumors can evade immune-mediated cell killing, eventually leading to disease recurrence and progression [22,34]. The unique ecosystem of TME can often dramatically impact tumor response to therapy and patient clinical outcomes.

### 2.2. Interaction Between the TNBC Genomic Subclass and the TME

Understanding how the nature of the TME varies between the different TNBC subtypes can help to develop unique therapeutic approaches specific to each subtype and potentially improve patient outcomes. Interestingly, each TNBC subtype has unique genetic abnormalities; for instance, in the BL-1 subtype, many DNA repairs (ATR-BRCA), cell cycle, and proliferation genes are dysregulated. In the BL-2 subtype, numerous kinase signaling pathways (EGFR, MET, NGF, Wnt/B-catenin, etc.) gluconeogenesis, and myoepithelial markers are dysregulated, in MSC-like sub-type, cell motility, differentiation, EMT growth factor-related signaling is abnormally regulated, and in the MSC stem-like TNBC subgroup, angiogenesis genes are dysregulated. In the LAR subtype, TNBC alteration of AR and luminal gene expression are differentially modulated. Importantly, in the IM subtype, many immune cell process-related genes (*CTLA4*, *IL2*, and *IL7*), immune-modular pathways, and antigen processing/presentation are dysregulated. Although, all subtypes of TNBC demonstrate considerable numbers of gene mutations, for example, *TP53*, *CTNNA1*, *DDX18*, *HUWE1*, *NFKBIA*, *APC*, *BRAF*, *MAP 2 K4*, and *RB1*, and these mutations are predominately observed in the IM TNBC subtype [35].

The subtype-specific gene expression patterns and alteration of the downstream pathways result in unique microenvironments and resultant immune consequences. For example, the BL-1, BL-2, and M TNBC subtypes show an “immune cold” phenotype (poor adaptive and innate immune responses) [36], because of the limited infiltration CD8+ T-cells in BL-2 and M subtypes [37,38]. MSLCs sub-group TME is mainly characterized by the presence of neutrophils, eosinophils, interdigitating dendritic cells (iDCs), NK cells, mast cells, (innate immune cells), and high M2-TAMs [36,39]. LAR TME exhibits the presence of innate immune cells, such as mast cells, and iDCs, predominantly [36]. Lower levels of TILs, an increased number of CD4+ and CD8+ T-cells, reduced Tregs and cycling T-cells, and the activation of MDSCs and IFN-γ signaling were also reported in LAR-TME [40]. Notably, IM TNBC subtype TME is referred to as an immune-rich and immune-hot TME, not only due to the high representation of adaptive immune cells such as CD8+ T cells, CD4+ T-cells, B-cells, γδ T-cells, NK cells and FOXP3+ cells [36,41], but also due to the high expression of immune-related hub genes including *BIRC3*, *CSF2RB*, *BTN3A1*, *GZMB*, *HCLS1*, *GIMAP7*, *LCP2*, and *SELL* [42]. Indeed, except IM subtypes, all other TNBC subtypes (LAR, M, and BL2) TME are immunosuppressive. Here, we comprehensively discuss the varying immune cell types within the TNBC TME and their various roles in tumorigenic or tumor suppression and molecular pathway regulation.

### 2.3. Immune Invasion Within the TNBC TME

Primary immune cells found within the TNBC TME are TILs, particularly T cells [43], and a high infiltration of TILs is associated with neoadjuvant chemotherapy (NAC) response in TNBC [44]. Studies, to date, indicate that CD4+ and CD8+ T cells have opposing roles in breast cancer progression and outcomes. CD8+ T cells are primarily involved in tumor killing and improved prognosis, whereas intra-tumoral CD4+ T cells have negative prognostic effects on breast cancer patient outcomes [45]. In physiological conditions, the infiltration of TILs into the TNBC TME has not been studied in detail, whereas several other factors can modulate TILs infiltration in TNBC. For example, a recent animal study suggests that in mice fed with vitamin C, CD8+ T cell infiltration is enhanced in TNBC xenograft tumors by the suppression of PD-L1 expression [46] and the overexpression of LIV1, zinc (Zn) transporter, in TNBC, has been found to modulate infiltration CD4+/CD8+ T cells in TNBC tumors [46]. Further, the direct-immunohistochemistry analysis revealed that CD4+ high/CD8+ low expression was observed in lower LIV1 expression, and CD4+ low/CD8+ high protein expression is associated with high LIV1 expression [47]. Increased circulating and tumor-infiltrating B lymphocytes (TIL-B) and CD20+CD27+IgD− isotype-switched B lymphocytes were observed in TNBC patient’s blood, and a higher presence of TIL-B was associated with improved clinical outcomes [48]. Single-cell and bulk RNA sequencing data in TNBC samples revealed that B cell markers such as *ZBP1*, *SEL1L3*, *CCND2*, *TNFRSF13C*, *HSPA6*, *PLPP5*, *CXCR4*, *GZMB*, and *CCDC50* are predominantly associated with predicting prognosis and response to immunotherapy in TNBC patients indicates infiltration of B cells in TNBC [49]. A cohort study indicates that, in metastatic TNBC (mTNBC), high levels of CD4, CD8, and protein expression, and a high ratio of CD4/FOXP3, CD8/CD163, and CD8/FOXP3 improve one-year overall survival in de novo of mTNBC patients [50] and stromal lymphocytic infiltration increased prognostic value in TNBCs patients [51].

Polymorphonuclear PMN-MDSCs and monocytic M-MDSCs are immunosuppressive, they inhibit immune response mediated by T cells, B cells, and NK cell activity [52]. Since they originated from bone marrow precursors [53], MDSCs are further classified into (CD11b^+^CD14^+^HLA-DR^−/low^ CD15^−^) monocytic-MDSCs and (CD11b^+^CD15^+^ LOX1+CD14^−^) granulocytic-MDSCs based on marker expression [54,55]. After infiltration in tumor cells, MDSCs regulate immune responses leading to poor clinical outcomes [56]. PMN-MDSCs showed a higher expression of genes associated with the cell cycle, autophagy, G protein signaling, and the CREB pathway, whereas M-MDSCs showed an upregulation of several genes related to the neutrophil functions and chemokines receptor 1 (CXCR1). There is a significant overlap between the gene signatures of PMN-MDSCs and M-MDSCs (*IL1B*, *ARG2*, *CD84*, and *WFDC17*) [57], suggesting that both kinds of MDSCs exert similar immunosuppressive effects with different mechanisms [58]. Numerous cytokines and chemokines such as IL-6, IL-1β, G-CSF, M-CSF, GM-CSF, macrophage migration inhibitory factor (MIF), and TGF-1 were present in the TME which attract MDSCs accumulation at tumor sites [59]. A recent study suggests that inhibition of biosynthesis of MDSCs by dihydroorotate dehydrogenase (DHODH) inhibitors suppressed MDSCs production from early-stage myeloid progenitors that enhanced myeloid maturation leads to immune activation (CD8+T cell) and increased antitumor and antimetastatic activities and immune checkpoint inhibitor sensitivity (PD-L1 based) [58,60]. Higher levels of MDSCs accumulation in the orthotopic 4T1 mammary carcinoma tumors were observed, and natural killer T (NKT) cell activation via α-GalCer-loaded dendritic cells (DCs) decreased the immunosuppressive activity of MDSCs [61]. Although the presence of a higher number of MDSCs in TME creates immunosuppressive effects, the dynamic role of MDSCs under therapy in TNBC TME is not yet clear.

Macrophages are myeloid cells involved in pathogen and dead cell elimination and stimulation of the action of other immune cells. TAMs are derived from circulating blood mononuclear cells and differentiate into macrophages after exuding tissues [62]. Generally, macrophages are two types of classically activated macrophages 1 (M1) and alternatively activated macrophages 2 (M2), and their classification is also tissue-specific. The recruitment of TAMs depends on the surrounding TME, and infiltration of TAMs in TNBC TME modulates immune suppression, cancer cell phenotype, tumor development, metastasis, and angiogenesis which contribute to poor prognosis in cancer patients [63]. In TME, TAM M1 inhibits tumorigenesis whereas TAM M2 enhances tumorigenesis/drug resistance [64]. M1 is associated with pro-inflammatory and M2 with anti-inflammatory phenotypes due to their distinct gene expression profiles and M2 is further classified as M2a, M2b, M2c, and M2d [65]. TAM-M1 expresses several cytokines IL-1B, IL-6, IL-8, IL-12, IL-18, TNFα, etc. M2a expresses IL-10, IL-13, IL-1RA, TGF-β and others; M2b expresses IL-10, IL-1b, IL-6 and others; M2c expresses IL-10, TGF-β, and others; and M2d expresses IL-10, IL-12, TGF-β, TNFα, etc. The cytokine expression in M1 and M2 depends on the inflammatory status of TME and in most situations TAMs create immunosuppressive TME [66].

Higher levels of TAMs are found in TNBC TME compared to other types of breast cancers [67]. In TNBC, TAMs participate in tumor occurrence and development and metastasis, therefore TAMs serve as potential biomarkers for prognosis prediction [62,68]. TNBC tumor cells secrete several factors, including macrophage colony-stimulating factors (M-CSF) and IL-6, which drive macrophages toward M2 polarization [69,70]. M2 CD163+ and CD68+ macrophages are more abundant in TNBC/basal-like breast cancer than luminal types [70]. M2 CD68+ macrophages secrete IL-6 and CCL5, which correlate with poor prognosis [71,72]. Regardless of the ER, PR, and HER2 status or the use of the HER2-targeted trastuzumab drug, the high count of M2 CD163+ TAM was associated with a poor outcome in HER2+ breast cancer patients [73]. The high expression of M2 CD163+ in invasive breast cancer (IBC) is associated with increased cell proliferation index and larger tumor size [74]. On the contrary, a recent study suggests that higher densities of CD163+ macrophage infiltration TNBC tumors improved overall survival and breast cancer-specific survival independently in invasive TNBC [75]. Stromal mammary gland tissue-resident macrophages (MGTRMs) also play an important role in early TNBC before angiogenesis. Local depletion of MGTRMs by corpus luteum (CL) reduced tumor recurrence, and distant metastases, and improved chemotherapeutic output [68]. Mechanistically, TAM-M2 stimulates epithelial–mesenchymal transition (EMT) and cancer stem cell (CSC) properties in TNBC by the activation of CCL2/AKT/β-catenin signaling [76]. Interestingly, the transition of M2 to M1 was observed in TME after exposure to *Taraxacum mongolicum* dandelion extract. Dandelion extract increased the expression of M1-like marker TNF-α, IL-8, and iNOS, but reduced M2-like marker IL-10, CD206, Arginase-1, and TGF-β [77]. The high infiltration of TAMs in tumor tissues is associated with poor patient prognosis; therefore, macrophage-targeting therapy such as TAM depletion/TAM differentiation/TAM activation is required for tumor cell elimination/phagocytosis in TME. These strategies are urgently needed for better outcomes for TNBC patients.

The participation of activated and heterogenous CAFs in TME modulates TNBC tumor development [78]. These fibroblasts decrease anti-tumor immunity and promote cell proliferation, migration, invasion, and neo-angiogenesis by expressing the extracellular matrix (EMC) protein and creating an immunosuppressive microenvironment [79,80]. CAFs by the activation of transforming growth factor beta (TGF-β) a cytokine, may promote TNBC development and progression [81]. The in vitro study demonstrated that CAFs produced high levels of interleukin-chemokine expression, including IL-6, IL-8, CXCL1, CXCL3, and TGF-β when co-cultured with breast cancer cells [82]. Mechanistically, myeloid cells express CXCL16 that activates CAFs which infiltrate more myeloid cells and fibroblasts in TNBC [83]. CAF-related G protein-coupled receptor 34 (GPR34) expression is high in TNBC, suggesting that GPR34 serves as a prognosis biomarker in TNBC in response to immunotherapy in TNBC patients [84], whereas CAF subtypes (CAF+ and CAF-) modulate overall survival (OS), immune cell infiltration, and immunotherapy response differently in TNBC. The CAF- subtype associated with TNBC is linked to overall survival (OS), and more immune cells than the CAF+ subtype and representative pathway analysis revealed that the CAF- subtype enriched in immune-related pathways and CAF+ subtype with extracellular matrix pathways [84].

TANs are another important cell population present in TME and can be activated under various conditions (Chemo/RT) and infiltration TANs in TME may be immunosuppressive [85]. A TAN is divided into two phenotypes, N1- and N2-type, and, like macrophages (M1 and M2), N2-type promotes tumor growth. N2 can be converted into N1-type by inhibiting/blocking TGF-β or enhancing interferon (IFN) production [86]. Moreover, TNBC tumor cells secrete granulocyte-macrophage colony-stimulating factor (GM-CSF), TGF-β, and CXCR2 which stimulates TANs to release tumor suppressor M, promotes angiogenesis, and improves tumor cell infiltration or recruitment of neutrophils in TNBC [87,88,89]. In particular, the CXCL-8/CXCR-2 axis plays a crucial role in the recruitment of N2 neutrophils to the TME, further contributing to immunosuppressive conditions [90]. Targeting this pathway offers potential therapeutic strategies to modulate TAN activity and improve outcomes for TNBC patients.

NK cells derived from bone marrow hematopoietic stem cells modulate TME, particularly in triple-negative breast cancer (TNBC). Their innate ability to target and destroy cancer cells without prior antigen-specific immunity makes them vital in anti-tumor responses. NK cells recognize tumor cells through mechanisms such as MHC-I downregulation, a hallmark of many cancers, including TNBC [91]. Upon activation, NK cells release cytotoxic molecules such as perforin and granzyme to induce apoptosis and secrete cytokines like TNF-α and IFN-γ. These cytokines enhance anti-tumor immune responses by promoting the recruitment of other immune cells and modulating the immune landscape. Additionally, NK cells secrete chemokines such as CCL1, CCL2, CCL3, CCL4, CCL5, and CXCL8, which contribute to the recruitment of T cells and macrophages into the TME, and tumor cell destruction [92]. However, TNBC often creates an immunosuppressive TME that limits NK cell efficacy. Strategies employed by TNBC to evade NK cell-mediated responses include upregulating immune checkpoint molecules like PD-L1 and shedding ligands for NK cell-activating receptors such as MICA and MICB, further impairing their cytotoxic function [93,94]. Overcoming these immunosuppressive mechanisms through therapies like immune checkpoint inhibitors (ICIs) or cytokine stimulation (e.g., IL-2 and IL-15) has shown the potential to restore NK cell activity in TNBC [95,96]. Advances in chimeric antigen receptor-engineered NK (CAR-NK) cells have demonstrated significant promise in TNBC. CAR-NK cells targeting HER1, engineered with catalase, have shown efficacy in mitigating oxidative stress within the TME, enhancing their cytotoxic potential and preventing postoperative local and distant relapses of TNBC tumors [97]. Additionally, CAR-NK cells targeting other antigens like EGFR and CD19 have shown preclinical success in enhancing TNBC tumor lysis, highlighting their versatility as a therapeutic strategy [98,99]. Combining CAR-NK cell therapy with immune checkpoint blockade or TME-modulating agents could amplify therapeutic responses, offering hope for TNBC patients resistant to conventional treatments.

CAAs are immunomodulators and they are present in breast cancer TME. CAAs can provide high energy to tumor cells by supplying different metabolites such as ketones, fatty acids, pyruvate, and lactic acid [100]. CAAs secrete chemokine ligands CCL2 and CCL5, interleukin-1 (IL-1), interleukin-6 (IL-6), tumor necrosis factor-α (TNF-α), and vascular endothelial growth factor (VEGF) [101,102,103], and promote cell aggressiveness, tumor progression, migration, and chemotherapy resistance [104]. By partial induction of CCL5, adipocytes increased the invasiveness of TNBC MDA-MB-231 cells [103]. Importantly, CAA-PD-L1 expression impaired the anti-tumor function of CD8+ T cells and inhibition of CAA-PD-L1 by lipoinhibitors increasing immunotherapy sensitivity [105]. In addition, hesperidin PLGA nanoparticles (Hesperidin, a natural phenolic compound) inhibit CCL2, elevate ADPN secretion in CAAs, decrease the recruitment of M2 macrophages, and potentiate the efficacy of a PD-1 in TNBC [106]. CAAs and CAA-derived CXCL8 cytokine modulate tumor growth, EMT, metastasis, and tumor immunity suppression [107]. CAA-secreted CXCL8 suppressed CD4+ T and CD8+ T immune cell infiltration and modulated CD274 upregulation in TNBC [107]. The study further revealed that targeting the CXCL8 pathway and PD-1 inhibition synergistically increased the tumor immune response and TNBC tumor suppression [107]. The possible roles of different immune cells in TNBC-TME are summarized in Table 1.

However, understanding the alteration in TME during therapy is important, in the next sections, we discuss how RT can modulate the TME and how this may be exploited to improve radiation sensitivity in TNBC.

## 3. Radiation and the TME

### 3.1. Impact of RT on the TME

**Figure 2:**
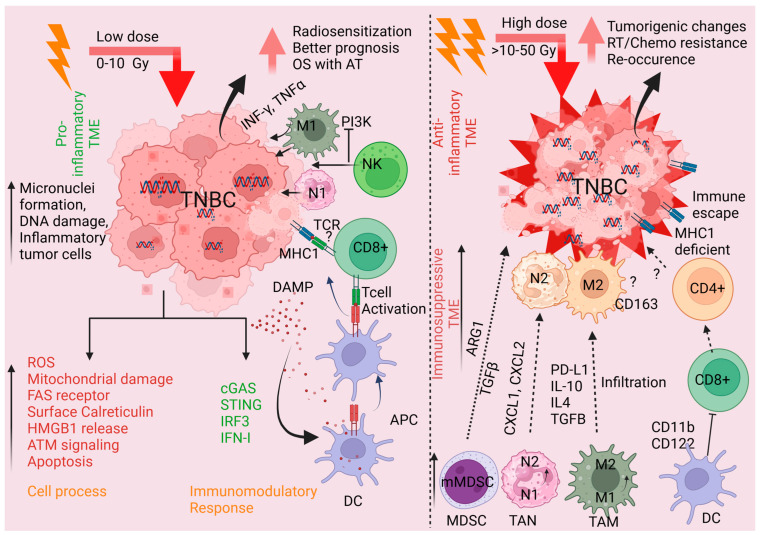
The schematic model represents the possible role of RT in TNBC TME. Low-dose RT exposure may induce DNA damage, micronuclei formation, and upregulation of pro-inflammatory signaling in TME that leads to increased ROS production, mitochondrial damage, and cell apoptosis. RT exposure activates ATM signaling and increases calreticulin expression on the tumor cell surface and the release of HMGB1 and DAMP from tumor cells. RT activates cGAS/STING/IRF3/INF-1 immunomodulatory signaling, DC and cytotoxic T cells, and targets tumor cells. Higher infiltration of TAM-M1 and TAN-N1 induces an innate immune response which leads to increased radiosensitization and better prognosis/overall survival in TNBC patients with adjuvant therapy. On the other hand, increasing RT creates immunosuppressive TME by increasing infiltration of TAM-M2, TAN-N2, and MDSC leading to tumor immune escape and RT/chemoresistance and re-occurrence and metastasis. The schematic model was generated using BioRender. OS—overall survival; AT—Adjuvant therapy.

Generally, RT is believed to convert immunologically “cold” tumors into “hot” tumors, leading to improved loco-regional control. RT can also improve systemic response to cancer immunotherapy [108,109]. RT makes tumors “hot” by enhancing antigen presentation and tumor immunogenicity, promoting immune cell infiltration into tumor sites, and increasing the priming of phagocytic and cytotoxic cells near tumor-associated antigens [108,109,110]. RT also induces micronuclei formation to activate nucleic acid sensors in cytoplasmic compartments, which can in turn activate the cyclic GMP-AMP synthase-stimulator of interferon genes (cGAS-STING) pathway [111] and the expression of type I interferon (IFN-I) [112]. Simultaneously, RT upregulates FAS (death receptor), MHC class I, translocation of calreticulin to tumor cell surfaces, and increases the release of HMGB1 from dying tumor cells (Figure 2). RT-mediated induction of these cellular processes in TME enhances the maturation of dendritic cells, and the secretion of cytokines and chemokines around the tumor enhances tumor-infiltrating lymphocyte (TIL) trafficking [113,114]. Furthermore, repetitive or high-dose RT-induced DNA double-strand breaks (DSBs) activate ataxia–telangiectasia mutated (ATM), interferon signaling, and the innate immune system [112,115,116] (Figure 2). To receive RT-mediated immunotherapeutic impacts, intact immunity, type-I interferon production, and CD8+ T cell infiltration are essential [117].

**Figure 3:**
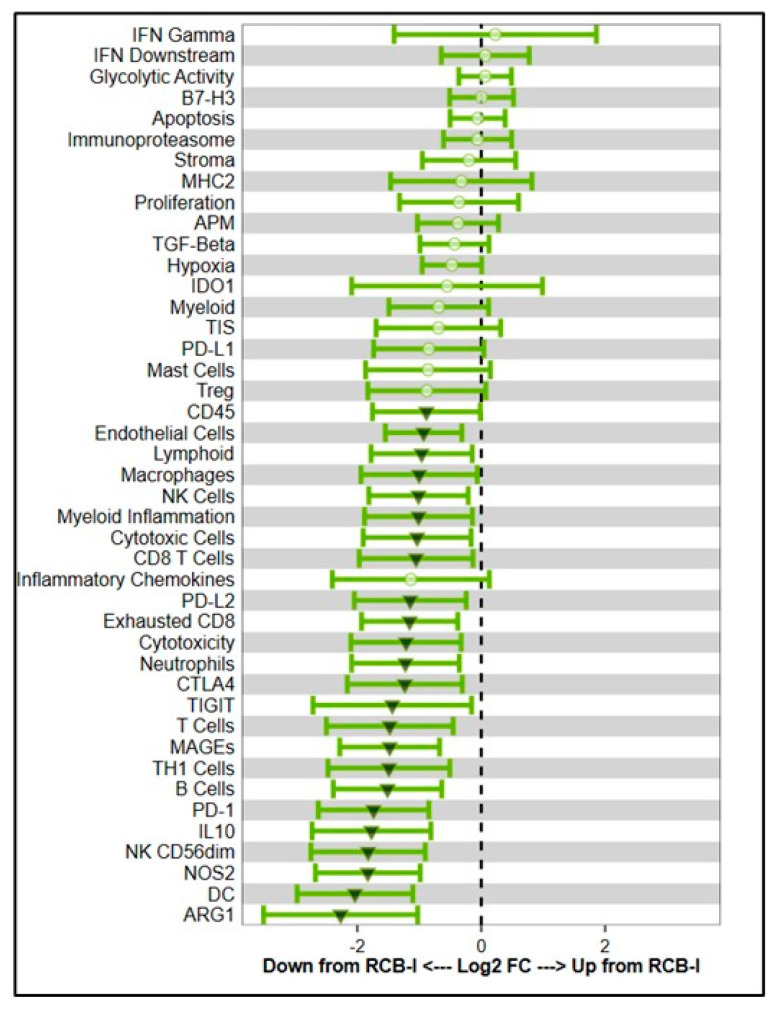
Increased residual cancer burden after RT was associated with reduced immune activation. NanoString RNA sequencing was performed [124] after RT exposure of breast cancer patients and residual cancer burden (RCB-II)-associated pathways and reduced levels of multiple gene expression associated with the antigen presentation and immune activation were presented. Arrow indicates downregulation RCB associated pathways.

In addition to immune promotion impacts, RT can also induce the secretion of immunosuppressive cytokines and chemokines such as TGF-β and IL-10, promoting local and systemic immunosuppression, as well lead to lymphopenia via lymphocyte cell death (Figure 2). RT also induces the production of MDSCs, and conversion of M1 into M2 TAMs, and Tregs, which can lead to immune escape, recurrence, and tumor progression [55,118,119]. RT leads to cellular DNA damage and the release of anti-inflammatory cytokines like IL-1, IL-6, and granulocyte-macrophage colony-stimulating factor (GM-CSF), facilitating MDSCs recruitment and accumulation in the TME (Figure 2). These MDSCs impair anti-tumor immunity and create immunosuppressive TME by releasing reactive oxygen species (ROS), nitric oxide (NO), and arginase, which inhibit T cell proliferation and effector functions. [120,121]. Furthermore, RT enhances MDSC-mediated immune evasion by activating signaling pathways, including STAT3, NF-κB, and COX-2. These pathways drive the suppressive capabilities of MDSCs, enhancing their role in promoting immune resistance and supporting tumor survival. Notably, hypoxia-induced factors like HIF-1α further augment MDSC function by increasing the expression of immunosuppressive molecules such as iNOS and arginase while simultaneously upregulating PD-L1 on MDSCs, contributing to immune escape mechanisms [122]. Targeting MDSCs to mitigate RT-induced immunosuppression has shown promise. For instance, inhibitors targeting STAT3 and arginase or strategies blocking the COX-2 pathway can reverse MDSC-mediated immunosuppression and improve the efficacy of RT [55,123]. Preclinical studies suggest that combining RT with targeted therapies enhances T cell infiltration and anti-tumor activity, offering a potential therapeutic avenue for overcoming immune resistance in TME. Recently, we analyzed residual cancer burden via expression genes associated with antigen presentation and immune activation in breast cancer patients using NanoString RNA sequencing after RT exposure. Our preliminary data demonstrated that RT reduced the levels of multiple genes associated with antigen presentation and immune activation including cytolytic NK CD56dim cells, antigen-presenting dendritic cells, neutrophils, B cells, NK cells, the expression of the interferon-gamma signaling mediator, nitric oxide synthase, IL-10, PD-1, CTLA4, TIGIT, ARG1, and others (Figure 3) suggesting that RT can cause immunosuppression TME. These findings emphasize the dual nature of RT in promoting anti-tumor immune responses while also facilitating immune resistance.

In the next sections, we discuss how radiation-mediated impacts on the TNBC TME may be exploited to improve tumor control and patient outcomes.

### 3.2. Potential Means of Therapeutically Exploiting RT-Mediated Impacts on TNBC TME

Compared with other breast cancer subtypes, the TME of TNBC is quite different because of the higher infiltration of TILs, TAMs, high expression of VEGF and other cytokine and chemokines [125], poor interaction between T cell PD-1 and tumor cell PD-L1, [126] and low response to ICIs monotherapy [127]. RT plays a dual role in the TME by directly killing cancer cells through DNA damage and sometimes promoting or suppressing anti-tumor immunity. RT affects multiple cellular pathways, re-modulates the TME, and increases immunotherapeutic sensitivity (Figure 2). RT-induced tumor cell DNA breaks leading to the genomic instability that triggers tumor cell apoptosis, the release of high mobility group box 1 protein (HMGB1) and ATP [128,129], and the translocation of calreticulin on the tumor cell surface (eat-me signal) [129,130,131]. On the other hand, activated dendritic cells (DCs) present in TME bind to HMGB1 and promote antigen cross-presentation and T-cell priming (Figure 2). RT triggers the release of chemokines (CXCL10 and CXCL16) from tumor cells to recruit effector T cells to the tumor site, thereby enhancing the anti-tumor immune response [113]. RT dose, RT type, and tumor signaling (cytokine and chemokine levels) modulate macrophage reprogramming in TME [132]. A low RT dose (1–10 Gy) recruits M1 (pro-inflammatory) and a high RT dose (>10 Gy) recruits M2 (anti-inflammatory) [133,134] and the recruitment of M2 has a higher degree of radiation resistance compared to the M1 [133]. The aggregation and increase (2.2- to 2.88-fold) of macrophages was observed in breast tumors’ TME after RT, specifically 14 to 73% iNOS+ M1 macrophages [135]. RT released HMGB1 from tumor cells that induce high TNF-α and low levels of IL-10 secretion from M1-type macrophages that exert drastic anti-tumor activity [136]. An increased CXCR6 expression by TNBC cells regulates CD8+T differentiation, upregulation of cytotoxic markers, and the intercellular communication of immune cell subtypes is associated with better clinical outcomes in patients given with adjuvant radiotherapy and immunotherapy [137]. Prostaglandin reductase 1 (PTGR1)-mediated immune evasion mechanisms are related to the activation of M2 and CD8+T cells, by which the target gene regulates immune cell impact on TNBC progression [138]. EGFR expression and the RT-mediated trans-endothelial migration of CAR-T cells activate the NF-κB pathway to induce the expression of intercellular adhesion molecule 1 (ICAM1) in TNBC cells providing long-lasting antitumor effects [139]. Mice TNBC xenograft models suggest that RT-mediated NK cell migration and penetration into the primary tumor site reduced tumor burden and growth [140]. Inhibition of the PI3Kαδ and PI3Kγδ or PI3K-AKTmTOR pathway by the reduction in TNBC tumor hypoxia-sensitized TNBC to RT-mediated DNA damage suggests that the synergetic effects of RT and anti-PD-L1 therapy in CT26 murine colorectal carcinoma cells, TNBC cell and TNBC mouse models [141,142]. RT and anti-PD-L1 antibody or CTanti-CTLA-4 (9H10 monoclonal antibody) administration in mice reduced TNBC tumor growth and lung metastasis in animals, indicating that RT with blockade of PD-1 or CTLA-4 may be the effective strategy against TNBC metastasis [143,144]. RT exerts immunosuppression impacts on TME and RT-mediated DNA damage and its impact on immune modulation in TNBC was reviewed recently [145], and the possible role of RT in different tumors of TME was also reported recently [146]. The role of RT in immune regulation for TNBC TME is summarized in Table 2.

Bone niche TME: Importantly, stage 4 TNBC is a highly metastatic cancer that has spread beyond the breast and nearby lymph nodes to distant organs like the liver, lungs, brain, and bones. A “bone niche” TME consists of osteoblasts, osteoclasts, mesenchymal stem cells, endothelial cells, immune cells (T cell, macrophages, MDSCs), cancer-associated fibroblasts, adipocytes, pericytes, and proteins like extracellular matrix (ECM), collagen and osteopontin [155]. There are three main components responsible for tumor growth in bone niche TME, i.e., osteoblasts (responsible for bone formation), osteoclasts (involved in bone resorption), and a chemokine, CXCL12, mainly involved in attracting TNBC cells [156]. Moreover, MDSCs contribute to an immunosuppressive milieu in bone niche TME that facilitates tumor establishment, regrowth, and metastasis. In this context, RT directly affects the function of cancer cells, bone marrow stromal cells, immune cells, and blood vessels which lead to tumor destruction. As in other TME, RT dose and treatment regimen also can potentiate radiation resistance in the bone niche TME [157], and CAFs, and mesenchymal stem cells, can contribute to radioresistance by producing growth factors and ECM components that protect tumor cells [158]. TNBC bone metastasis is associated with poor prognosis with median survival rates limited to several months. Further studies are, therefore, required to understand the interplay between RT and the bone niche TME, which can in turn guide the development of combination therapies targeting specific components of the TME to enhance RT efficacy [159].

Tumor TME Models: Since the in vitro monolayer cultured tumor cell line-based model is unable to mimic the microenvironment of tumors, to recapitulate the radiation response of the TME, several in vivo preclinical models have been recently postulated [160,161]. A combination of in vitro three-dimensional (3D) models and preclinical in vivo models might be a more reliable and robust approach to effectively investigate clinically relevant anticancer therapies. Integration of 2D and 3D models, spheroids and organoid cultures, tissue bioengineering, in vivo animal models, and single cell sequencing technologies are required to reveal the heterogenicity and complexity within the tumor and TME [161]. To characterize the TME, several vivo models have been developed recently including syngeneic models, xenograft models, and orthotopic models. In the orthotopic model, implantation of patient-derived cell xenografts (PDX) into animals is performed at the corresponding anatomic site [160]. PDX strategy is often better because it allows tumor development in a relevant environment and provides important information about anticancer treatment efficacy that mimics the disease process in humans [162]. The role of RT in remodeling the TME of TNBC has not yet been addressed in detail. Therefore, recently, we developed a syngeneic TNBC mouse model and analyzed immune invasion in TME. Our preliminary data suggest that CD163+ TAMs contribute to radioresistance in TNBC tumors [163]. Further studies are required to elucidate the mechanisms underlying radiation response in TNBC and whether novel molecularly targeted therapies can tip this balance toward increased radiation response and improved clinical outcomes.

### 3.3. Radiosensitization of TNBC

RT-mediated tumor sensitization approaches must be considered for better outcomes in TNBC patients. Although RT causing resistance/reoccurrence in TNBC is common, it is important to understand how RT targeting gene expression and signaling pathways in TNBC are crucial for chemotherapy and immunotherapy management. RT not only induces DNA damage but also modulates several cellular pathways in tumor cells. Recently, Li et al. reported that Elongin B (ELOB), a transcription elongation/regulation of gene expression protein, enhances RT efficacy in TNBC [164]. CRISPR-Cas 6-mediated depletion of ELOB reduced mitochondrial oxygen consumption rate (OCR) and enhanced radiosensitivity in TNBC cells—the study further suggesting that high expression of ELOB is associated with poor prognosis in TNBC patients who have received radiation therapy [164]. Similarly, the depletion of X-ray repair cross-complementing protein 4, (XRCC4), a DNA repair protein involved in non-homologous end joining (NHEJ), contributes to radio sensitization in TNBC. Comet assay revealed that XRCC4 knockdown increased radiation-mediated DNA damage and decreased cell growth in TNBC-MDA-MB-231 cells. The author pointed out that, despite downregulation and variations in XRCC4 expression in TNBC patients, a high expression of XRCC4 is associated with poor progression-free survival after radiotherapy [165]. The inhibition of Polo-like Kinase 4 (PLK4) by CFI-400945 drug significantly enhances the anticancer effects of radiotherapy (RT) compared to drugs or radiation exposure on TNBC cells alone [166], and the depletion of PLK4 by siRNA or the inhibition of PLK4 by CFI-400945 or Centrinone B and exposed with RT, induced antiproliferative effect and increased radio sensitization by overamplification of centrioles in TNBC cells [167]. Radiosensitization in TNBC tumor xenografts with tumor growth delay and decreased overall survival was observed in BUB1 (cell cycle Ser/Thr kinase) ablation of TNBC cells suggesting that BUB1 is a possible target and responsible for radiosensitization [168]. Mechanistically, BUB1 ablation inhibited the repair of radiation-induced DNA double-strand breaks (DSBs), recruitment of phospho- and total-DNAPK, and KAP1 to chromatin indicating that BUB1 is indispensable in the activation and recruitment of non-homologous end joining (NHEJ) proteins to DSBs [168]. Increased DNA damage was found after inhibition of threonine tyrosine kinase inhibition (TTKi) and RT, compared to RT alone, indicating TTK inhibition impaired DNA damage repair mechanisms, and homologous recombination (HR). However, the reintroduction of wild-type TTK rescued both radioresistance and HR repair efficiency, suggesting that TTK is an important RT resistance target in TNBC [169]. RT-induced DNA double-strand breaks and activates RAD50, a DNA repair protein (Mer11-Rad500Nbc1 complex), and activation of RAD50 repairs DNA break that lead to radioresistance; however, silencing of RAD50 by siRNA nanoparticles, RT enhances cell apoptosis in TNBC [170]. TNBC radioresistant (TNBC/RR) cells when exposed to 2-Thio-6-azauridine (TAU) a repurposed antiviral drug, inhibit cell viability and migration and induce apoptosis by transcriptional downregulation of CD151 (T cell activator) indicates that CD151 may be the therapy response (TAU+RT) marker in TNBC [149]. Earlier reports suggest that the deletion/inhibition or overexpression of various gene targets, particularly oncogenes in TNBC, activate the PI3K/AKt signaling and induce radiotherapy resistance [129]. Moreover, the 45% to 75% TNBC overexpresses the epidermal growth factor receptor (EGFR) gene. Blocking of EGFR-TK domain by DNA alkylation inducer ZR-BA1 induced radiation mediated double-strand breaks and impaired DNA repair in MDA-MB-468 TNBC cells and 4T1 of mouse TNBC cells [171]. Further, Maternal Embryonic Leucine Zipper Kinase (MELK) is also a biomarker for radio-resistance, a study suggests that high expression of MELK in breast cancer tissues and TNBC, and expression of MELK is significantly associated with radioresistance [172] and inactivation of MELK (genetically and pharmacologically) increased radiation sensitivity in vitro and inhibits tumor growth in vivo [172].

In addition, several microRNAs (miRs) participate in various regulatory mechanisms of TNBC. In TNBC, miR expression is differentially regulated and associated with tumorigenesis or tumor suppression, for example, by targeting transmembrane 4 L6 family member 1 (TM4SF1), miR-206 affects cell migration and invasion in MDA-MB-231 TNBC cells [173], miR-340 also affect cell invasion and metastasis by targeting and inhibiting Rho Kinase 1 (ROCK1) [174], and miR-124 target STAT3 and STAT3 signaling and downregulates cell proliferation and invasion [175]. An earlier study demonstrated that in TNBC miR-142.3p target Lysyl oxidase (LOX) (an enzyme involved collagen and elastin crosslinking), Hypoxia-inducible factor 1-alpha (HIF1α) and integrin subunit alpha 5 (ITGA5), and causes chemoresistance [176], and miR-21 target PTEN and increased TNBC cell proliferation and invasion [177]. Since miRNAs modulated cell proliferation, migration, invasion and metastasis, drug resistance, and apoptosis, using cohorts of three datasets, Hong et al. analyzed the predictive prognostic signature of miRs expression for TNBC [178]. Notably, eight miRs; miR-139-5p, miR-10b-5p, miR-486-5p, miR-455-3p, miR-107, miR-146b-5p, miR-324-5p, and miR-20a-5p expression is predicted in TNBC patients after post-surgical relapse. The expression of miR-139-5p, miR-10b-5p, and miR-486-5p are down-regulated and miR-455-3p, miR-107, miR-146b-5p, miR-324-5p, and miR-20a-5p are up-regulated and interestingly, miR-139-5p expression correlates with disease-free survival and TNM stage in TNBC patients [178].

Radio sensitization in TNBC is also modulated by miRs expression, for example, miR-27 is highly expressed in TNBC MDA-MB-435 and MDA-MB-231 cell lines and expression of miR-27 targets CDC27 which leads to radiation resistance and thus regulates the cell proliferation and radiation sensitivity in TNBC cell lines [179]. Recently, radiation response-related miRs expression has been analyzed in TNBC MDA-MB-231 and T47D breast cancer cells and the data suggest that T47D cells are more sensitive to radiation than MDA-MB-231. The expression of miR-16-5p and miR-23b-3p is associated with radiation response, and radiation-increased phosphorylation of ATM, TP53, and CDK1 and increased expression of RAD51 and γH2AX DNA damage markers. Bioinformatics analysis revealed that miR-16-5p targets cell cycle-related genes and may be responsible for longer overall survival of breast cancer patients [180]. Using PubMed, EMBASE, and Web of Science data, To et al. revealed that 35 miRs are significantly associated with RT in TNBC in which 21 are downregulated, 13 are upregulated, and 2 had a double-side expression. The miR-21, miR-33a, miR-139-5p, and miR-210 are related to the TNBC patient outcome after RT, and miR-7, miR-27a, miR-155, miR-205, miR-211, and miR-221, are regulated in response to RT [181]. Radiosensitive miRs’ expression profile must be considered before adjuvant radiation therapy (RT). Indeed, these studies suggest that apart from DNA damage, RT modulates the expression of several oncogenes, signaling pathways, and miRNAs and sensitizes TNBC to chemotherapy or immunotherapy. The role of RT that targets TNBC-associated factors and radiosensitization is summarized in Table 3.

### 3.4. Novel Clinical Trials Assessing Radiosensitization of TNBC

**Figure 4:**
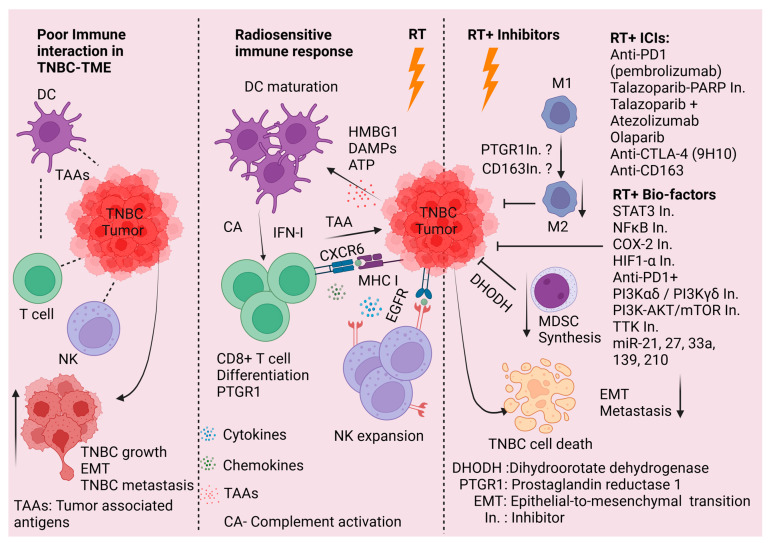
The schematic model represents the possible RT role in adjuvant therapy for TNBC. During TNBC growth, poor interaction and immune invasion enhance TNBC tumorigenesis and progression. A lack of antigen presentation by dendritic cells (DCs) is unable to activate cytotoxic T cells and NK cells hence TNBC gains EMT and metastatic phenotypic potential. On the other hand, RT induces immune-responsive TME by the indication of HMBG1, DAMPs, ATP, etc., from tumor cells that enhance DC maturation, able to capture tumor surface antigen for CD8+ T cell presentation. RT modulates CD8+ T cell and NK cell differentiation and expansion by releasing several tumor-related cytokines, chemokines, and other factors leading to immunogenic attacks. On the other side, RT induces infiltration of macrophage M2 and MDSC into TME and creates immunosuppressive TEM by inducing anti-inflammatory signaling. To enhance the immunotherapeutic impact, inhibition of M2 activity (anti-CD163) and MDSCs synthesis, use of immunotherapy-based anti-PD1 anti-CTLA4 antibodies, ICIs, PARP inhibitors, and tumor cell targeted transcription faction and signaling pathway inhibitors potentiate RT efficacy. The use of RT in adjuvant therapy (chemotherapy and immunotherapy) curtails EMT and the metastatic potential of TMBC which leads to increased overall survival in TMBC patients. The schematic model was generated using BioRender.

RT is considered to be an immunomodulatory modality. RT can execute DNA breaks and mutations, generate genomic instability in tumor cells, and increase immunotherapeutic antigen presentation [186]. Conversely, RT can induce immunosuppression within the TME that may decrease the infiltration of effector immune cells to the tumor site [187]. RT, in combination with immunotherapy (blockade of CTLA-4 and PD-1/PD-L1), exerts synergistic antitumor impacts [188]. Additionally, RT not only inhibits tumor cells but also shows inhibitory effects outside of the irradiation field [189]. Using ICIs in early-stage TNBC [140], in combination with optimal RT doses, may offer a promising treatment approach for this aggressive malignancy (Figure 4).

Numerous clinical trials investigating the potential synergy of using RT plus ICIs or targeted inhibitors for TNBC to improve clinical responses are underway (Figure 4). The TNBC RADIOPARP phase 1 trial recommends the use of PARP inhibitor Olaparib 200mg twice/day with RT [151] similar to the NCT01618357 phase 1 trial for node-positive, residual breast cancer [152]. PD-1/PD-L1 and CTLA-4/B7-1/B7-2 based ICIs breast cancer clinical data have been reported recently [190,191]. In phase 2 clinical trial (NCT02730130), the use of monoclonal immunoglobulin pembrolizumab, a PD-1 inhibitor, plus RT is recommended for metastatic TNBC who were unselected for PD-L1 [153]. Moreover, a recent phase 2 Study (NCT04690855) evaluated the efficacy and safety of talazoparib, RT, and atezolizumab in germline BRCA 1/2 negative patients with PD-L1+ metastatic TNBC. Both talazoparib, a PARP inhibitor, and radiation (XRT) independently increase PD-L1 expression on the tumor cell surface resulting in enhanced sensitivity to PD-L1 inhibitor, atezolizumab [154]. This trial predicts the combination of talazoparib, XRT, and atezolizumab to re-sensitize metastatic TNBC tumors to immunotherapy and promotes a durable tumor-specific response with lower toxicities compared to traditional chemotherapy [154].

## 4. Conclusions and Future Directions

TNBC is a highly aggressive subtype of breast cancer lacking ER, PR, and HER2 expression, with a propensity for disease recurrence and distant spread. Despite aggressive treatment approaches utilizing surgery, chemotherapy, radiation therapy, and immunotherapy, TNBC patients often show poor prognosis following treatment. In this review, we discuss the biological heterogeneity of the TNBC subtype and the dynamic changes that are elicited by radiation within the TME. Indeed, RT has a dual effect on TNBC tumors, i.e., sensitization for cell death or acquiring radio–chemo and immunoresistance. TNBC TME is inherently immunosuppressive due to the poor interaction of tumor cells with the surrounding T cells and NK cells. RT can alter the dynamics of the TME by releasing cytokines, chemokines, DAMPs, and HMBG1 from TNBC tumors, triggering DC cell maturation and activation of T cells and NK cells (Figure 4). On the other hand, RT can invite macrophages (M2) and MDSCs to the tumor vicinity to create an immunosuppressive TME that promotes immune escape. Inhibition of TAMs (M2) or MDSCs and targeting and managing anti-inflammatory signaling pathways before radiation delivery may improve RT response. In addition, beyond PD-1/PD-L1 and CTLA-4-directed immunotherapies, T-cell immunoglobulin and mucin domain 3 (TIM-3), lymphocyte activation gene 3 (LAG-3), adoptive cell therapy (ACT), cytokine therapies, and cancer vaccines need to be investigated in combination with RT to enhance the immune response against TNBC tumors. Furthermore, the utility of radio-immunotherapy should be assessed in the oligometastatic and advanced TNBC [192]. Finally, to gain a better understanding of TNBC behavior and to develop novel approaches to improve radiation response in this aggressive malignancy, more patients with TNBC should be encouraged to participate in clinical trials that involve RT.

## Figures and Tables

**Table 1:** The Possible Roles of Immune Cells in TNBC TME.

Immune Cells	Role in TNBC TME	Ref.
TILs	High infiltration of TILs is observed in TNBC-TME and is associated with neoadjuvant chemotherapy (NAC) response.	[44]
TILs	Stromal lymphocytic infiltration increased prognostic value in TNBC patients.	[51]
TILs	Infiltration of B cells and B cell marker expression predominantly associated with predicting prognosis and response to immunotherapy in TNBC patients.	[49]
TILs	T-cell infiltration and a high ratio of CD4/FOXP3 and CD8/CD163 proteins improve one-year overall survival in metastatic TNBC patients.	[50]
TILs	Increased CD8+T cell infiltration in TNBC xenograft tumors suppresses PD-L1 expression when mice fed vitamin C and LIVI overexpression modulate this process.	[46]
MDSCs	Inhibition of MDSCs biosynthesis enhances immunotherapy efficacy by myeloid maturation and activation (CD8+ T cell) in BC.	[60]
MDSCs	Immunosuppressive MDSCs accumulation in the TNBC 4T1 cell tumor mouse model was observed and NKT cell activation via DCs decreased MDSCs immunosuppressive activity.	[61]
Macrophages	Higher levels of TAMs are found in TNBC TME and macrophage colony-stimulating factors (M-CSF) and IL-6 drive macrophages toward M2 polarization and infiltration.	[67,69,70]
Macrophages	Higher numbers of M2 CD163+ and CD68+ macrophages are present in TNBC/basal-like breast cancer compared to luminal types.	[70]
Macrophages	Infiltration of higher densities of CD163+ macrophage TNBC tumors improved OS and BC-specific survival independently in invasive TNBC.	[75]
Macrophages	By activation of CCL2/AKT/β-catenin signaling, TAM-M2 stimulates EMT and cancer stem cell (CSC) properties in TNBC.	[76]
CAFs	The presence of CAFs in TNBC TME promotes TNBC progression by the activation of TGF-β.	[81]
CAFs	Myeloid cells mediated expression of CXCL16 activates CAFs and promotes fibroblast infiltration in TNBC TME.	[83]
CAFs	High expression of CAF-related G protein-coupled receptor 34 (CAF-GPR34) in TNBC patients serves as a prognosis biomarker in response to immunotherapy.	[84]
TANs	TNBC tumor cells release GM-CSF, TGF-β, and CXCR2 stimulate TANs to release tumor suppressor M, promote angiogenesis, and improve tumor cell infiltration or recruitment of neutrophils in TNBC-TME.	[87,88,89]
NK cells	By downregulation MHC-I, NK cells recognize TNBC tumor cells and ICIs, and cytokine stimulation restores NK cell activity in TNBC-TME.	[91,95,96]
NK cells	CAR-NK cells targeting HER1, engineered with catalase in TNBC-TME, modulate cytotoxic potential and prevent postoperative local and distant relapses of TNBC tumors.	[97]
CAAs	By the induction of CCL5, CAAs increased the invasiveness of TNBC MDA-MB-231 cells.	[103]
CAAs	Exposure of hesperidin to CAA inhibits CCL2, elevates ADPN secretion, reduces recruitment of M2 macrophages, and potentiates the efficacy of PD-1 in TNBC TME.	[106]
CAAs	In TNBC-TME, CAA meditated secretion of CXCL8 suppressed CD4+ T and CD8+ T and upregulating CD274 suggests targeting the CXCL8 and PD-1 inhibition synergistically increased the tumor immune response.	[107]

**Table 2:** RT Impact on TNBC TME.

Agents	Target	Mechanisms	Ref.
RT	TILs, TAMs	Higher infiltration in TNBC TME	[125]
RT	CXCR6, CD8+ T cells	Elevated CXCR6 regulates CD8+T differentiation leads to superior response to adjuvant radiotherapy and immunotherapy in TNBC.	[137]
RT	EGFR	Migration of CAR-T cells in TME, activates the NF-κB, and induces ICAM1 that regulates antitumor effects in TNBC	[139]
Hypofrac. RT (HFRT)	MDSCs	Combined HFRT, and CXCR2 blockade inhibits MDSCs infiltration in TNBC tumors and increases the efficacy of CAR-T cells	[147]
Radiolabeled biomolecule(RB) + RT + anti-PDL1 and-CTLA4 (CP)	Macrophages MDSCs	RB + RT + CP suppressed macrophages and MDSCs, infiltration in TNBC tumors that contributes to immune escape and tumor relapse.	[148]
TAU+ RT	CD151 (T cell activator)	Transcriptional downregulation of CD151	[149]
RT	NK cells	NK cell migration and penetration into the primary TNBC tumor reduced tumor burden and growth	[140]
PI3Kαδ Inhibition. +RT	PI3Kαδ and PI3Kγδ	Reduce TNBC tumor hypoxia and Antitumor Immune Effect of Anti-PD1 and RT sensitization	[141]
PI3KδγInhibition +RT	CD8+T	Potentiates effector CD8(+) T cell-dependent antitumor and abscopal effect after RT	[142]
RT + anti-PD-1	PD-1	Reduced TNBC tumor growth and metastasis in mice	[143]
RT + siRNA	PD-L1	TNBC 4T1 derived cell membrane (CM) coated PD-L1 siRNA-decorated Au/MnO_2_ nanosensitizer (R&F@Au/MnO_2_-CM) enhances radio-immunotherapy synergistically.	[150]
RT + anti CTLA-4	CTLA-4	Inhibit TNBC lung metastasis and increase survival in mice	[144]
Olaparib +RT	PARP	Improve clinical responses	[151,152]
Pembrolizumab +RT	PD-1	Immunosuppressive, and safe for mTNBC PD-L1 negative patients	[153]
TAlazoparib + RT	PARP	Increased tumor PD-L1 expression enhanced sensitivity to PD-L1 inhibitor, atezolizumab.	[154]

**Table 3:** Radiosensitivity and Related TNBC signaling.

Agents	Target	Mechanisms	Ref.
RT	Elongin B	Reduced mitochondrial oxygen consumption rate.	[164]
RT	XRCC4	Knockdown increased radiation-mediated DNA damage.	[165]
CFI-400945 + RT	Polo-like Kinase 4 (PLK4)	Increase antiproliferative/radio sensitization by overamplification of centrioles.	[166]
RT	BUB1 (cell cycle Ser/Thr kinase)	BUB1 deletion impaired RT-mediated DSBs repair by recruitment of phospho-, total-DNAPK, and KAP1 to chromatin.	[168]
RT	Threonine tyrosine kinase inhibition (TTKi)	TTK knockdown or inhibition reduces tumor growth in vivo.	[169]
RT	RAD50, a DNA repair protein	Silencing of RAD50 by siRNA nanoparticles, RT enhances cell apoptosis	[170]
ZR-BA1+ RT	EGFR	Induced DSBs and impaired DNA repair	[171]
OTSSP167 + RT	MELK	Inactivation/deletion of MELK increased radiation sensitivity and inhibited tumor growth	[172]
RT	miR-27	MiR-27 targets CDC27 and involves the radiosensitivity of TNBC cells	[179]
RT	FOS/Phospholipase C beta 1 (PLCB1)	RT mediates FOS/PLCB1-induced radioresistance by impairing CD8+ T cell activity and by the activation of PI3K/AKT signaling pathway.	[182]
RT	γH2AX and p53	Increased DNA damage in MCF-7 BC compared to the TNBC and CAFs extracted from the tumor tissue (TNBC subtype tumor) shows increased resistance to ionizing compared to luminal A tumors isolated.	[183]
RT + Regorafenib	Multi-kinase inhibitor	Regorafenib enhanced the radiosensitivity of TNBC but not MCF 10a normal breast cells.	[184]
RT	Cathepsin S (CTSS)	Inhibition of CTSS restores BRCA1 function and enhances RT-induced apoptosis of TNBC cells.	[185]
RT	miR-16-5p and miR-23b-3p	RT induces miR-16-5p in breast cancer but not in TNBC and is associated with radiation response	[180]
RT	miR-21, miR-33a, miR-139-5p, and miR-210	Associated with radiation response in TNBC patients	[181]

## Data Availability

Gene expression analysis was performed using the NanoString nCounter Immuno-Oncology 360 (IO360) panel (NanoString Technologies, US). Data quality control was conducted using nSolver Analysis Software (ver. 4.0) and NanoStringQCpro (ver. 1.14.0). The data that support this study’s findings are available on request from the corresponding author, D.S.
